# Medical Practitioners and Hospital Abuse

**Published:** 1904-11-19

**Authors:** J. Marshall


### EDITOR'S LETTER-BOX.

### MEDICAL PRACTITIONERS AND HOSPITAL ABUSE.

*To the Editor of* THE HOSPITAL.

SIR,—Allow me to congratulate you on your leading article on "Medical Practitioners and Hospital Abuse." As a general practitioner of twenty years' standing (six years in London) I have maintained for many years that huge sums of money are squandered yearly by our hospital authorities, under the guise of charity, in London notoriously so. The out-patient departments are principally responsible for this annual waste, and yet the hospital authorities in their doleful annual wail for more money have the audacity to state how many thousands have been treated as out-patients, when they know, or at least ought to know, that an enormous percentage of those patients have unworthily assisted them in swallowing up funds that a generous but uninformed charitable public had provided for the needy poor. I am glad to notice from your leading article that Battersea is to the front in actual reform. My remedy for hospital abuse is in a nutshell, very much as you put it. "Let no one be treated in the out-patient department (except emergency cases) without a recommendation by a doctor." Might I suggest that your leading article should be reprinted in pamphlet form and circulated amongst the Governors of the various hospitals? Yours etc.

Stepney. J. MARSHALL.

